# How well documented are testing strategies and outcome measurement methods in trials of tests? A comparison of reporting quality in test-treatment and monitoring RCTS

**DOI:** 10.1186/1745-6215-14-S1-P14

**Published:** 2013-11-29

**Authors:** Jac Dinnes, Lavinia Ferrante di Ruffano, Alice Sitch, Julie Parkes, Jenny Hewison, Doug Altman, Jon Deeks

**Affiliations:** 1 University of Birmingham, Birmingham, UK; 2 University of Southampton, Southampton, UK; 3 University of Leeds, Leeds, UK; 4 University of Oxford, Oxford, UK

## Background

Given the advantages of the randomised controlled trial (RCT) design for the evaluation of therapeutic interventions, it is tempting to assume that the same approach must be the gold standard for the evaluation of testing strategies. Such trials present considerable challenges, due to the complex nature of the decision–making process. To interpret how changes in testing strategies create observed effects, trials must pre–specify how test results should inform diagnostic and management decisions and treatment plans.

## Aim

To assess and compare reporting quality of testing strategies in test-treatment RCTs and RCTs of monitoring strategies.

## Method

Published RCTs were ascertained systematically for two separate projects:

1. Those evaluating any diagnostic test and measuring patient outcomes (CENTRAL 2009).

2. Those evaluating any strategy for monitoring for disease progression or recurrence (CENTRAL 2011).

Trial reports were appraised regarding documentation of interventions, and completeness of primary outcome reporting.

## Results

103 test-treatment RCTs and 58 monitoring RCTs were included. In test-treatment trials, the nature of the intervention was frequently unclear: 10% described the study intervention, 6% the control intervention. Outcome measurement methods were adequate enough to be replicated in 51%. These results will be compared to those for monitoring RCTs.

## Conclusions

Preliminary findings suggest that interpretation of effects observed in both test-treatment and monitoring RCTs are hampered by incomplete reporting of interventions and outcome assessment. Testing strategies require more detailed description of multiple decision–making components compared to other types of interventions. A new CONSORT extension may be required to improve standards.

